# Accuracy of the molecular diagnosis of duchenne and becker muscular dystrophy: A systematic review with meta-analysis

**DOI:** 10.1371/journal.pone.0345550

**Published:** 2026-07-21

**Authors:** Fabian A. Chavez-Ecos, Carlos Quispe-Vicuña, Marco Malaga, Niels Pacheco-Barrios, Andrely Huerta-Rosario, Victor Velasquez-Rimachi, Peggy Martinez Esteban, Carlos Alva-Diaz

**Affiliations:** 1 Grupo de Investigación Neurociencias, Metabolismo, Efectividad Clínica y Sanitaria (NEMECS). Universidad Científica del Sur, Lima, Perú; 2 Sociedad Científica de Estudiantes de Medicina de Ica, Facultad de Medicina Humana de Ica, Universidad Nacional San Luis Gonzaga, Ica, Perú; 3 Hospital Nacional Víctor Larco Herrera, Lima, Perú; 4 Instituto Nacional de Salud del Niño - San Borja, San Borja, Bolivia; 5 Servicio de Neurología. Departamento de Medicina y Oficina de Apoyo a la Docencia e Investigación (OADI), Hospital Daniel Alcides Carrión, Callao, Perú; University of Miami Miller School of Medicine: University of Miami School of Medicine, UNITED STATES OF AMERICA

## Abstract

**Introduction:**

Recently, Molecular diagnosis of Duchenne muscular dystrophy (DMD) and Becker muscular dystrophy (BMD) has become increasingly important in the management of these patients, with techniques such as multiplex ligation-dependent amplification (MLPA) and next-generation sequencing (NGS) coming to the fore. Therefore, this study aims to evaluate the diagnostic accuracy of MLPA, NGS, and the algorithm MLPA-NGS for confirmatory diagnosis of DMD/BMD.

**Methods:**

We systematically searched databases (PubMed, Embase, Scopus, Cochrane and Web of Science) until July 2025 for studies evaluating the diagnostic accuracy of MLPA and/or NGS testing in patients with clinical suspicion of DMD, considering multiplex PCR or biopsy as the reference test. A meta-analysis was performed using a random-effects model to estimate the sensitivity, specificity, and detection rate of each test. The QUADAS-2 tool was used to assess the risk of bias and the GRADE criteria were used to identify the certainty of evidence.

**Results:**

We included 10 studies (3786 patients) evaluating the use of MLPA and 14 studies (4333 patients) evaluating the use of NGS. For MLPA, the sensitivity was 0.80 (95%CI: 0.76–0.84; *I*^*2*^: 86%), the specificity was 0.93 (95%CI: 0.87–0.96; *I*^*2*^: 16%), and AUC 0.90 (CI-95%: 0.89–0.92). For NGS, the detection rate was 0.77 (95% CI: 0.61–0.87; *I*^*2*^: 94%). Furthermore, the detection rate increased to 0.97 (95% CI: 0.94–0.99; *I*^*2*^: 95%) when NGS was performed after MLPA. We observe a low risk of bias but with very low certainty in the estimations.

**Conclusions:**

In patients with clinical suspicion of DMD, the MLPA test is very good but with very low certainty. However, in these patients with a negative MLPA, adding the NGS test would allow improve the detection rate. Therefore, the sequential use of these tests could be considered in patients who persist in the clinical suspicion.

## Introduction

Duchenne Muscular Dystrophy (DMD) is a genetic disorder caused by a mutation in the DMD gene located on the short arm of the X chromosome, at locus Xp21.2-p21.1, which encodes the dystrophin protein found in muscle cells [1], which encodes the dystrophin protein found in muscle cells. Dystrophin’s role is to maintain the connection between the extracellular matrix and the cytoplasmic cytoskeleton [1]. Globally, the DMD birth rate ranges from 7 to 20 per 100,000 males approximately and is mainly expressed in males [2] causing delays in motor development and weakness in proximal muscles with difficulty standing and walking, frequent falls, and pseudohypertrophy of the calf muscle [3,4]. Diagnosis of DMD involves identifying specific mutations within the DMD gene [5]. The majority of cases are due to deletions (68%) and duplications (11%), with a smaller proportion caused by small-scale mutations (20%) such as insertions, splice-site mutations, nonsense mutations, and missense mutations4. Mutations may occur anywhere in the gene but are more concentrated between exons 45–55 and exons 2–10, respectively [3,4,6]. It is important to distinguish DMD from Becker Muscular Dystrophy (BMD), another dystrophinopathy caused by mutations in the same DMD gene. While both conditions share a similar genetic basis, BMD is generally characterized by a later onset and slower progression of muscle weakness compared to DMD. The mutations in BMD often result in a partially functional dystrophin protein, leading to less severe clinical symptoms. To detect mutations associated with DMD and BMD, various molecular techniques are used, including multiplex polymerase chain reaction (PCR-multiplex), multiplex ligation-dependent probe amplification (MLPA), and Next Generation Sequencing (NGS) [4,7,8].

PCR-multiplex has become the established diagnostic standard for detecting deletions in the dystrophin gene, with success rates ranging from 60 to 70% by amplifying the most commonly deleted exons. However, while PCR is reliable for identifying deletions, it has limitations in detecting duplications in most patients, and deletions in female carriers may be masked by the presence of a normal X chromosome [7,9,10]. In contrast, MLPA can detect deletions and duplications of medium size that are not usually detected by PCR or cytogenetic techniques, but it cannot detect small mutations [9,11]. Finally, NGS allows, for simultaneous sequencing of thousands or millions of fragments of DNA in a single process, to examine small genetic mutations in the dystrophin gene with or without large deletion or duplication, especially in non-coding regions [8,11]. These methods have become preferred by clinicians. However, the systematic analyses of the existent evidence are still pending. We aim to evaluate the diagnostic accuracy of MLPA, NGS, and the algorithm MLPA-NGS for confirmatory diagnosis of DMD/BMD.

## Methods

We performed a systematic review and meta-analysis following PRISMA guidelines [12], the extension version “Preferred Reporting Items for a Systematic Review and Meta-analysis of Diagnostic Test Accuracy Studies” (PRISMA-DTA) [12] (S1 Table in S1 File), and the Cochrane Handbook for Systematic Reviews of Diagnostic Test Accuracy [13]. The protocol was registered in PROSPERO (CRD42021243508). This review evaluated two research questions: 1) “In a patient with suspected DMD/BMD, what is the diagnostic accuracy of MLPA for the diagnosis of DMD/BMD?”; 2) “In a patient with suspected DMD/BMD, what is the diagnostic accuracy of NGS for the diagnosis of DMD/BMD?” In turn, the development of both questions allowed us to evaluate the diagnostic utility of combining both tests (MLPA-NGS algorithm).

### Eligibility criteria

The inclusion criteria are: 1) patients with clinical suspicion of DMD/BMD; 2) use MLPA and/or NGS, and the diagnosis confirmed by PCR multiplex, other molecular technique, or biopsy. Suspicion of DMD or BMD was usually based on characteristic clinical findings, such as progressive proximal muscle weakness, delayed motor milestones, gait abnormalities, among others. Similarly, biochemical suspicion was often supported by markedly elevated serum creatine kinase (CK) levels; additional indicators, such as a positive family history, electromyography (EMG) suggestive of a myopathic pattern, or abnormal dystrophin expression on muscle biopsy, further supported clinical suspicion prior to molecular confirmation. Our primary outcomes were: detection rate, sensitivity, specificity, positive and negative predictive values, and true and false positives and negatives for MLPA and/or NGS. The study design involved diagnostic studies with cohort, cross-sectional, and case series design. The exclusion criteria were case-control studies, case reports, letters to the editors, narrative reviews, systematic reviews, and diagnostic studies that do not report outcomes of interest.

### Data sources and searches

We searched in the following databases: PubMed, Scopus, Web of Science, and Embase for each diagnostic method MLPA and/or NGS until 1st October 2021 and was constantly updated until 5th July 2025. The search strategy free and Medical Subject Title (MESH) terms for “Muscular Dystrophy, Duchenne” or “Becker Muscular Dystrophy”, and “Molecular Diagnostic Techniques”. (Complete details of the search strategy appear in S2 Table in S1 File).

### Study selection

The search results were exported to EndNote X9 (Thompson and Reuters, Philadelphia, USA) to eliminate duplicate publications. Next, titles and abstracts of the retrieved publications were screened independently by two reviewers (MM and NP) to identify studies that potentially met the inclusion criteria of this review using the web application Rayyan (https://www.rayyan.ai), which is a free application that allows the management of data from multiple studies from various databases, to facilitate the selection of studies. Each screened publication, in the title and abstract phase as in the full-text phase, was made by duplicate by two reviewers (MM and NP) and any disagreement reached a consensus by a third reviewer (CAD).

### Data extraction and definitions

Two reviewers (MM and NP) independently extracted the relevant data using a standardized data extraction sheet. In case of disagreement, this was resolved by consensus between all authors or by a third reviewer (CAD). The following data was extracted for each study: publication date, first author country, type of study, inclusion/exclusion criteria, detection rate, sensitivity, specificity, true positive and negative, false positive and negative. When studies with the same population were identified, only the most recent or complete publication was included. If more data was required, we reached out to the corresponding author via email to request the necessary information. In cases where this was not feasible, the study was excluded from further analysis.

True positive (TP), true negative (TN), False positive (FP), and false negative (FN) was considered a diagnosis of DMD and non-DMD by MLPA, NGS, or algorithm MLPA-NGS confirmed by the reference standard (PCR multiplex or biopsy).

### Quality assessment of studies

Two authors (MM and NP) independently evaluated the risk of bias (RoB) using the QUADAS-II tool for DTA studies, which comprises four domains: patient selection, index test, reference standard, and flow and timing [14]. The authors assessed all domains in terms of RoB and the last three in terms of concerns regarding applicability, categorizing them as high, unclear, or low. However, for this review, specific criteria were established. In the “patient selection” domain, consecutive or random selection was not mandatory as long as case-control designs and improper exclusions were avoided. We included studies where patients had suspected diagnoses based on clinical presentations, biochemical changes, or genetic testing confirmatory. For the “index test” domain, all patients who were tested were included. The “reference standard” domain encompassed all patients who underwent genetic testing, muscle biopsy, or other genetic diagnostic procedures. For the “flow and timing” domain, we deemed the interval appropriate if the index and reference samples were collected at the same time.

### Certainty of evidence

The Grading of Recommendations Assessment, Development, and Evaluation (GRADE) method to evaluate the certainty of the evidence in diagnostic studies [15,16]. The risk of bias was assessed by pooling the weight of low-bias studies, imprecision by measuring the difference between sensitivity and specificity, heterogeneity with I^2^, and publication bias, with author-developed criteria described in S3 Table in S1 File. The certainty of evidence was characterized by using the online GRADE tool (http://gradepro.org).

In addition, for the calculation of the absolute effect per 1,000 patients analyzed, a prevalence of DMD of 96% was assumed from the studies included in this review.

### Statistical analysis

We considered sensitivity as TP/ participants with the disease (TP + FN), specificity as TN/ participants without the disease (TN + FP), positive predictive value as TP/ participants with a positive test (TP + FP), negative predictive value as TN/ participants with a negative test (TN + FN) and detection rate (DR) was defined as TP/ total patients in the study (TP + TN + FP + FN). Relationship between the ROC curve and diagnostic accuracy was classified as; 0.9–1.0 (excellent), 0.8–0.9 (very good), 0.7–0.8 (good), 0.6–0.7 (sufficient), 0.5–0.6 (bad), and <0.5 (test not useful) [17].

The statistical analysis will be carried out with the statistical package Rstudio version 4.3. The data were synthesized using a diagnostic meta-analysis, using a random effects model. To estimate the variance between studies (τ2) and calculate the combined effects, the Restricted Maximum Likelihood (REML) method [13] was specifically used to calculate the detection rate, sensitivity, and specificity. Accuracy data were logit-transformed to stabilize variance, and we used the Clopper-Pearson method to construct robust confidence intervals around these pooled estimates. To stabilize variances, we used pooled logistic regression (Plogit). A “leave one out” sensitivity analysis was used to understand the impact of each individual study on the overall estimate. Publication bias was assessed using the funnel plot asymmetry test and Egger’s and Deek’s tests. Heterogeneity was assessed using the chi-square and *I*^*2*^ statistics respectively. Heterogeneity will be defined as mild: I² < 40%, moderate: I² from 40% to 80%, and severe: I² > 80% [13].

## Results

### Study selection

**Fig 1:**
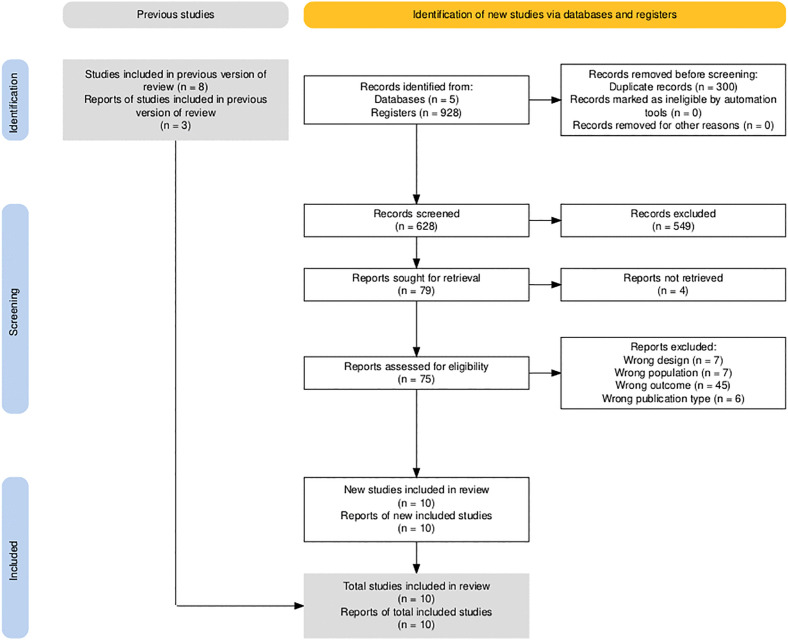
Selection process flowchart PRISMA 2020 for the question on MLPA.

**Fig 2:**
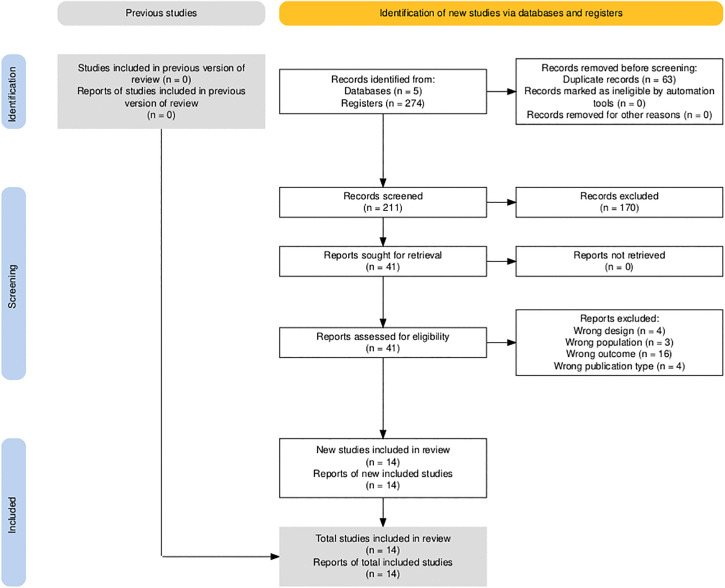
Selection process flowchart PRISMA 2020 for the question on NGS.

We identified 928 studies evaluating the use of MLPA. 300 studies were eliminated by duplication 628 studies were assessed for eligibility, and 10 studies were included. For NGS, 274 studies were identified, 63 were eliminated by duplication. Finally, 41 studies were reviewed a full text, and 14 studies were included for meta-analysis (Details of articles excluded are in S4 Table in S1 File). The PRISMA flowchart is reported in Fig 1 and 2.

### Study characteristics

Ten observational studies of MLPA (cross-sectional and cohort designs) had 3,786 participants [18–27]. Besides, nine studies were made in Asia’s countries [18,20–27], and only one in Latin America (Colombia) [19]. The index test for positive tests was the MLPA, and sequencing or biopsy was a gold standard for negative tests. For the NGS technique were 14 observational studies (cross-sectional and cohort design) with 4,333 participants [21–23,25,26,28–36], but NGS was employed only in 881 participants. 12 studies were made in Asia countries [21–23,25,26,30–36], and 2 in Latin America [28,29]. We used patients with clinical suspicion of DMD negative for the MLPA test for the algorithm MLPA-NGS. (Tables 1 and 2)

**Table 1:** Included studies on the use of MLPA in patients with clinical suspicion of DMD/ BMD.

Study	Year	Country	Type of study	Sample size/ Sampling type	Age*	Population	Reference test	Diagnostic sequence
Cho A et al. [18]	2017	Korea	Cohort	**◦** 507 **◦** Not random	17 ± 4	**◦** Male patients with genetically confirmed as DMD	Sanger sequencing	◦ The diagnostic process for DMD began with MLPA and then proceeded to muscle biopsy for MLPA-negative cases. ◦ After confirming dystrophinopathy, the direct Sanger sequencing was used for molecular diagnosis in MLPA-negative patients
Garcia-Acero et al. [19]	2018	Colombia	Cross-sectional	**◦** 62 **◦** Not random	9.0 ± 5.67	**◦** Male patients with clinical suspicion of DMD	NGS	In cases in which the MLPA did not detect deletions or duplications, gene sequencing was performed.
Guo R et al. [20]	2015	China	Cross-sectional	**◦** 613 **◦** Not random	NR	◦ Male patients diagnosed based on clinical symptoms and biopsy. ◦ Carrier patients were included in the study population	Sanger sequencing	In cases in which the MLPA was negative, gene sequencing was performed.
Kong X et al. [21]	2019	China	Cohort	◦ 1051 **◦** Not random	NR	◦ Patients with clinical suspicion; increase SCK level and abnormal EMG. **◦** Carrier patients were included in the study population	Sanger sequencing or NGS	In cases in which the MLPA was negative, NGS was performed.
Polavarapu K et al. [22]	2019	India	Retrospective cohort	◦ 804 ◦ Not random	Children	◦ Suspected cases of dystrophinopathy	NGS	In cases in which the MLPA was negative, NGS and/or muscle biopsy were performed.
Tomar S et al. [27]	2019	Singapore	Cross-sectional	**◦** 145 **◦** not random	NR	◦ Male patients with diagnosis by family history, clinical features, increased SCK level, EMG, and muscle biopsy	NGS sequencing	In cases in which the MLPA was negative, NGS was performed.
Wang DN et al. [23]	2017	China	Cohort	**◦** 128 ◦ Was included all records of patients with suspected DMD of the First Affiliated Hospital of Fujian Medical University from February 2013 to May 2016.	NR	◦ Patient with confirmed diagnosis of DMD by muscle biopsy y/o molecular tests	PCR	In cases in which the MLPA was negative, PCR was performed.
Wang DN et al. [23]	2019	China	Cross-sectional	◦ 70 ◦ Not random	3.47 ± 2.97	**◦** Patients with clinical suspicion; increase SCK level; myopathic abnormalities but normal peripheral nerve conduction velocity on EMG; and a positive family history with DMD ◦ Carrier patients were included in the study population	NGS	In cases in which the MLPA was negative, NGS was performed.
Zamani G et al. [25]	2020	Iran	Cohort	◦ 314 ◦ Was included all participants from the registries of Children’s Medical Center	15.26 ± 6.47	**◦** Patients with neuromuscular disability evaluated by physical examination increased SCK level, EMG and muscle biopsy.	NGS sequencing	In cases in which the MLPA was negative, NGS was performed.
Zhong J et al. [26]	2016	China	Cross-sectional	◦ 92 ◦ Was included all children with a suspected of DMD/BMD from a Pediatrics Department for Neuromuscular from 1 January 2011–1 November 2015	NR	◦ Patients with diagnosis by clinical presentation, family history, increase SCK level and calf muscle biopsy.	NGS sequencing	In cases in which the MLPA was negative, NGS was performed.

Age*: The age was expressed as mean ± SD (Standard Deviation) years; NR: Not reported; DMD: Duchenne muscular dystrophy; BMD: Becker Muscular Dystrophy; MLPA: Multiplex Ligation dependent Probe Amplification; SCK: serum creatine kinase; EMG: electromyography; NGS: Next-generation sequencing

**Table 2:** Included studies on the use of NGS in patients with clinical suspicion of DMD/ BMD.

Study	Year of publication	Country	Type of study	Sample size (NGS*)/ Sampling type	Age*	Population characteristics	Reference test	Diagnostic sequence
Alcantara-Ortigoza MA et al. [28]	2019	Mexico	Cross-sectional	• 72 (40) • Not random	11.25 ± 6.3	◦ Male patients with clinical suspicion of dystrophinopathy, but with normal mPCR results	Muscle biopsy	• NGS was applied to MLPA negative patients. It was not applied to all patients • Insufficient data is provided to assess the joint use of MLPA and NGS
De Almeida PAD et al. [29]	2017	Brazil	Cross-sectional	• 177 (52) • Was included all patients with a molecular diagnosis of the DMD/BMD of eight neuromuscular and genetics reference centers March 2015 and August 2016.	NR	◦ Patients with molecular diagnosis. Patients diagnosed by muscle biopsy or immunohistochemistry were not considered	NGS	• NGS was applied to MLPA negative patients • The index test was considered the gold standard
Kong X et al. [21]	2019	China	Cohort	• 1051 (223) • Not random	NR	◦ Patients with clinical manifestations, history of gastrocnemius pseudohypertrophy, increased SCK level, and abnormalities in EMG ◦ Carriers were included in the study population	Sanger sequencing	• NGS was applied to MLPA negative patients
Kumar S et al. [30]	2020	India	Cross-sectional	• 961 (246) • Was included all suspected DMD/BMD male patient of a healthcare center between 2006–2013	NR	◦ Patients with clinical suspicion but with negative mPCR results	NGS	• NGS was applied to MLPA negative patients. • The index test was considered the gold standard.
Okubo M et al. [31]	2016	China	Cross-sectional	• 67 (30) • Not random	NR	◦ Patients with different mutations like duplications, deletions, insertions, splice regions and missense mutations. ◦ Age: Not specified	Sanger sequencing	• NGS was applied to MLPA negative patients. • Insufficient data is provided to assess the joint use of MLPA and NGS.
Polavarapu K et al. [22]	2019	India	Retrospective cohort	• 804 (78) • Not random	NR	◦ Suspected cases of dystrophinopathy.	NGS	• NGS was applied to MLPA negative patients. • The index test was considered the gold standard.
Singh B et al. [32]	2017	India	Cross-sectional	• 18 (18) • Not random	3-29	◦ Patients with clinical features of DMD/BMD	NGS	• NGS was applied to MLPA negative patients • The index test was considered the gold standard • Insufficient data is provided to assess the joint use of MLPA and NGS
Tallapaka K et al. [33]	2019	India	Cross-sectional	• 510 (14) • Not random	7.25 ± 1.16	◦ Male patients with DMD phenotype	NGS	• NGS was applied to MLPA negative patients • The index test was considered the gold standard
Wang D et al. [24]	2019	China	Cross-sectional	• 70 (19) • Not random	3.47 ± 2.97	◦ Patient with clinical suspicion; increased SCK level; myopathic abnormalities but normal peripheral nerve conduction velocity on EMG; and a positive family history with DMD.	Sanger sequencing	• NGS was applied to MLPA negative patients
Wei X et al. [34]	2014	China	Cross-sectional	• 87 (34) • Not random	NR	Patients with diagnosis by immunohistochemical staining of dystrophin in muscle biopsies	Sanger sequencing	• NGS was applied to MLPA negative patients • Insufficient data is provided to assess the joint use of MLPA and NGS
Yamputchong P et al. [35]	2020	Thailand	Cohort	• 70 (32) • Was included all patients with a diagnosis of DMD of the pediatric neuromuscular clinic between 2017–2019.	12.0 ± 4.43	◦ Patients with diagnosis of DMD by clinical characteristics and increased SCK level	Sanger sequencing	• NGS was applied to MLPA negative patients
Yang Y et al. [36]	2019	China	Cohort	• 100 (22) • Not random	NR	◦ Patient with diagnosis of DMD/BMD based on SCK level, muscle biopsy, electromyography, electrocardiogram, progression of the disease, and family history.	Sanger sequencing	• NGS was applied to MLPA negative patients
Zamani G et al. [25]	2020	Iran	Cross-sectional	• 314 (46) • Was included all participants from the registries of Children’s Medical Center	15.26 ± 6.47	◦ Patients with neuromuscular disability and clinical suspicion; increased SCK level; abnormal electromyography findings; and presence of normal dystrophin in muscle biopsy.	NGS	• NGS was applied to MLPA negative patients
Zhong J et al. [26]	2016	China	Cross-sectional	• 92 (27) • Was included all children with a suspected of DMD/BMD from a Pediatrics Department for Neuromuscular from 1 January 2011–1 November 2015	NR	◦ Patient with DMD diagnosis by physical examination, electrodiagnostic tests, SCK, and muscle biopsy.	Sanger sequencing	• NGS was applied to MLPA negative patients

NGS: Next-generation sequencing; NGS*: Participants to whom the targeted NGS gene panel was applied; Age*: The age was expressed as mean ± SD years; NR: Not reported; mPCR: multiplex polymerase chain reaction; SCK: serum creatine kinase; EMG: electromyography; DMD: Duchenne muscular dystrophy; BMD: Becker Muscular Dystrophy

### Diagnostic utility

**Fig 3:**
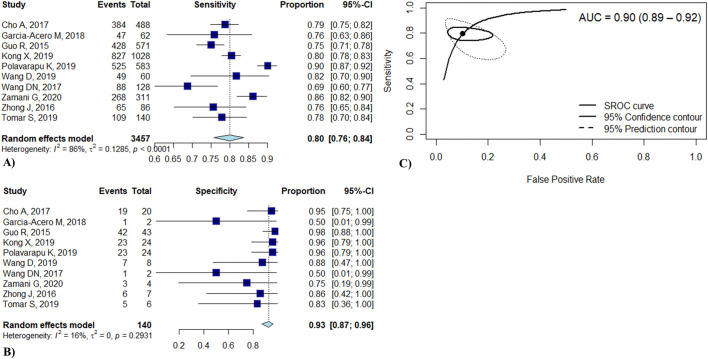
Meta-analysis of the diagnostic characteristics of the Amplification with Multiple Ligand Dependent Probes (MLPA), for the diagnosis of DMD/BMD in patients with clinical suspicion. a) Sensitivity; b) Specificity; c) ROC curve.

For MLPA the sensitivity and specificity were 0.80 (CI-95%: 0.76–0.84; *I*^*2*^: 86%) and 0.93 (95% CI: 0.87–0.96; *I*^*2*^: 16%) respectively; and AUC 0.90 (CI-95%: 0.89–0.92) (**Fig 3**).

**Fig 4:**
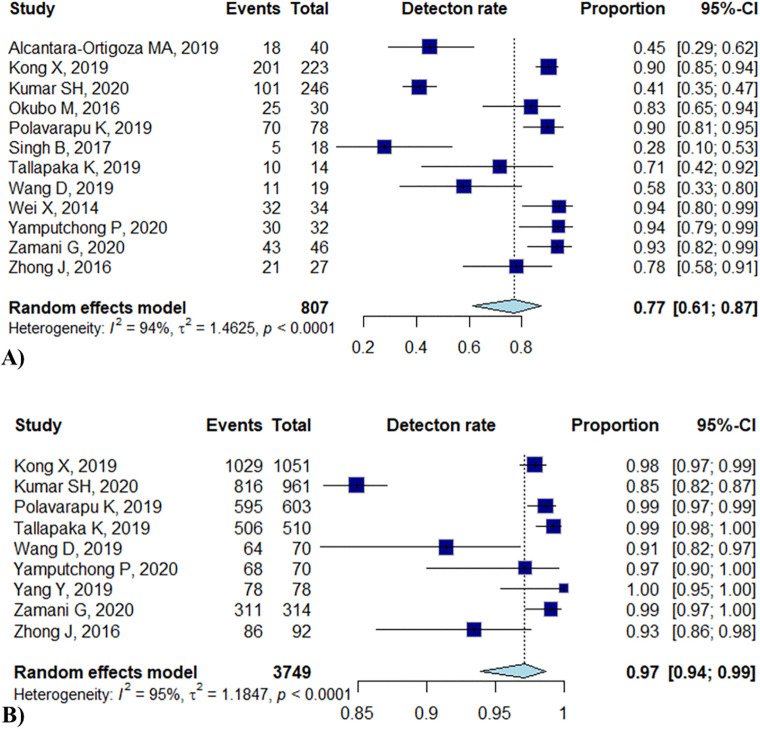
Meta-analysis of the detection rate of the studies included of the molecular test for patients with clinical suspicion of DMD/BMD negative for the MLPA. a) Meta-analysis of the detection rate of the NGS test for patients with clinical suspicion of DMD/BMD negative for the MLPA test; b) Meta-analysis of the detection rate of the MLPA + NGS algorithm for patients with clinical suspicion of DMD/BMD negative for the MLPA test.

The detection rate for NGS was 0.77 (95% CI: 0.61–0.87; *I*^*2*^: 94%) and for the algorithm MLPA-NGS, the detection rate was 0.97 (95% CI: 0.94–0.99; *I*^*2*^: 95%) (**Fig 4a and 4b**).

In the hypothetical scenario of a 1,000-person cohort, 768 (729–806) and 192 (153–230) patients may have been diagnosed with and without DMD, respectively. However, 37 (34–38) and 3 (2–5) patients may have been incorrectly diagnosed (**Table 3**).

**Table 3:** Summary of Findings according to GRADE certainty assessment.

№ of participants (studies)	GRADE certainty assessment					Effect (95% CI)	Absolute Effect per 1,000 patients tested (95% CI)	Certainty of the evidence
	Risk of bias	Indirectness	Inconsistency	Imprecision	Publication bias			
MLPA used to diagnosis DMD in patients with clinical suspect of DMD/BMD*								
3786 patients (10 studies)	Not serious	Not serious	Very serious^a^	Not serious	Serious^b^	Sensitivity 0.80 (0.76 to 0.84)	TPos: 768 (730–806)	⨁◯◯◯ Very Low
							FNeg: 192 (154–230)	
3786 patients (10 studies)	Not serious	Not serious	Not serious	Not serious	Serious^b^	Specificity 0.93 (0.87 to 0.96)	TNeg: 37 (35–38)	⨁⨁⨁◯ Moderate
							FPos: 3 (2–5)	
Detection rate of the NGS test for patients with clinical suspicion of DMD/BMD negative for the MLPA test								
3915 patients (12 studies)	Very serious^c^	Not serious	Very Serious^a^	Serious^d^	Not serious	Detection rate 0.77 (0.61 to 0.87)	TPos: 739 (586–835)	⨁◯◯◯ Very Low
							FNeg: 221 (125–374)	
Detection rate of the MLPA + NGS algorithm for patients with clinical suspicion of DMD/BMD negative for the MLPA test								
3671 patients (9 studies)	Not serious	Not serious	Very Serious^a^	Not serious	None	Detection rate 0.97 (0.94 to 0.99)	TPos: 931 (902–950)	⨁⨁◯◯ Low
							FNeg: 29 (10–58)	

**CI:** confidence interval; **TPos**: True Positive; **FNeg**: False Negative; **TNeg**: True Negative; **FPos**: False Positive

**GRADE Working Group grades of evidence**

**High certainty:** we are very confident that the true effect lies close to that of the estimate of the effect.

**Moderate certainty:** we are moderately confident in the effect estimate: the true effect is likely to be close to the estimate of the effect, but there is a possibility that it is substantially different.

**Low certainty:** our confidence in the effect estimate is limited: the true effect may be substantially different from the estimate of the effect.

**Very low certainty:** we have very little confidence in the effect estimate: the true effect is likely to be substantially different from the estimate of effect.

Explanations

^a^Two levels of certainty were decreased by presenting I^2^ > 80%.

^b^One level of certainty was decreased for presenting publication bias

^c^Two levels of certainty were decreased by less than 50% of studies have low risk of bias

^d^95% CI range is greater than 20%.

*Calculated prevalence: 96%

In the hypothetical scenario of a cohort of 1,000 persons, in which NGS was used, 739 (586–835) and 221 (125–374) patients may have been correctly and incorrectly diagnosed with DMD, respectively; however, the evidence is very uncertain (**Table 3**).

In the hypothetical scenario of a cohort of 1,000 persons, in which MLPA-NGS was used, 931 (902–950) and 29 (10–58) patients may have been correctly and incorrectly diagnosed with DMD (**Table 3**).

### Publication bias

Regarding the diagnostic accuracy of MLPA, the funnel plot distribution was visually asymmetrical, and Deek’s test provided evidence of small study effects (p-value < 0.001) (S1 Fig in S1 File). On the other hand, for the NGS detection rate, the funnel plot was visually symmetric, and Egger’s test did not provide evidence of small study effects (p-value = 0.078) (S2 Fig in S1 File).

### Sensitivity analysis

Sensitivity analysis revealed that, for both diagnostic accuracy and test detection rate, high heterogeneity did not change when studies were excluded one by one (S3 and S4 Figs in S1 File).

### Risk of bias assessment

For MLPA studies, we found that reference standard, index test, and flow and timing showed a low risk of bias. Regarding the patient selection criteria, 18.2% (2/11 studies) of the included studies presented a high risk of bias in the patient selection domain (S5 Table in S1 File).

Concerning NGS studies, the index test, reference standard, and patient selection presented a low risk of bias, because all patients had similar characteristics. However, flow and timing had a high risk of bias in the 57.1% (8/14 studies) of studies, because it was not specified (S6 Table in S1 File).

### Cost-effectiveness comparison between MLPA and NGS

In addition, a brief review was conducted to compare the cost-effectiveness of the two tests in order to better inform decision-making. The review found that MLPA stands out as a rapid (10–12 days) and cost-effective (US$200–700) option for detecting large genetic variants in routine laboratories. In contrast, NGS is more expensive (up to US$3,000+) and slower (2–8 weeks), but it is indispensable for identifying point mutations and also requires advanced bioinformatics infrastructure (S7 Table in S1 File).

### Certainty of evidence

In patients who utilized MLPA, we determined a “very low” and “moderate” certainty for sensitivity and specificity, respectively, because we decreased certainty due to very high heterogeneity for sensitivity and publication bias for specificity. In patients who used NGS as a screening test for DMD suspicion and were negative for the MLPA test, the detection rate was of “very low” certainty, because downgraded certainty by two levels due to less than 50% of studies with low RoB, two levels for very high heterogeneity, and one level by imprecision. Lastly, in MLPA + NGS we determined “low” certainty by inconsistency due to very high heterogeneity (**Table 3**).

## Discussion

### Summary of main results

For the diagnosis of DMD, we found for MLPA, the pooled sensitivity was 0.80 and the specificity was 0.93. For NGS, the detection rate was 0.77, which increased to 0.97 when the NGS test was performed after MLPA.

MLPA is a high-throughput method that determines the copy number of up to 50 genomic DNA sequences in a single multiplex PCR-based reaction, is easy to perform, requires only 20 ng of DNA sample, and does not require specialized equipment [37]. However, in the diagnosis of DMD it has been reported that negative results in this test must be confirmed by NGS testing [38]. NGS has become increasingly used in the last few years to diagnose multiple genetic disorders, as it can be used to sequence a gene, the exome, or the entire genome [39]. Targeted NGS has been used not only for diagnosing DMD patients but also for carrier and newborn screening. We found that NGS had a detection rate of 0.77 in MLPA-negative patients, and when combined with MLPA in a stepwise approach, this rate increased to 0.97. This approach has been recommended by the two consensus statements [1,40], but not by Colombian GRADE guideline [41]. This may be due to the highest incremental cost-effectiveness ratio (ICER) among studied interventions in a recent cost-effectiveness study [10].

### Agreements and disagreements with other reviews/evidence

The evidence indicates that deletion/duplication tests (e.g., MLPA, qPCR) detect pathogenic variants in 65–80% of cases, while sequence-based methods (e.g., NGS, Sanger) detect 20–35% [42]. This aligns with our findings showing a higher detection rate for deletion/duplication analysis. However, data for individual tests like MLPA or NGS remain unavailable, as previous reviews have not reported their specific diagnostic accuracy.

### Applicability of the evidence

Our findings could support the consensus and recommendations on the molecular diagnosis of DMD. A recent consensus on the diagnosis of DMD recommended an approach to identify mutations starting with MLPA or comparative genome hybridization followed by sequencing, either NGS or another technique [1]. This is similar to the recommendations given by the DMD Care Considerations Working Group [40]. The reasoning is that MLPA is able to detect both deletions and duplications [42], which correspond to a majority of patients with DMD [11]. This is in keeping with our findings, with a sensitivity of up to 80%, close to 75% of patients reported in a large cohort to have duplications and deletions. However, its cost remains a challenge, a cost-effectiveness analysis by Atehortúa et al. found that MLPA can be over 4 times more costly than multiplex PCR [10].

The high sensitivity and specificity reported for MLPA and NGS demonstrate that these tests have good utility in the diagnosis of DMD. In addition, it has previously been reported that MLPA can identify up to almost 6% more DMD mutations in those in whom multiplex PCR testing failed to detect intragenic deletions [43]. There is also the fact that the application of NGS to exclude negative samples following MLPA analysis raises the detection rate [21], and allows the evaluation of early-age patients with only clinical indicators (such as persistent hyperkalemia and myopathy on the electromyogram) but without defined clinical manifestations [23] to provide early diagnosis and treatment. However, the use of these tests requires specialized supplies and equipment [44] to perform them; in addition to the fact that they could be less cost-effective than other tests such as immunohistochemistry or Western blot [10]. Additionally, the personnel needed to perform and interpret these tests must have specialized training, leading to further costs for training. However, it’s important to note that these costs can vary considerably depending on the volume of tests, the availability of infrastructure, and technological advancements. In some cases, large-scale implementation of specialized tests might lead to economies of scale and reduce costs per test [10]. Despite this, the greater accuracy of MLPA and NGS over other tests would favor a higher risk-benefit ratio; especially in the detection of point mutations where the sequential use of MLPA followed by NGS becomes more useful [44].

The high molecular diagnostic accuracy found in this review, particularly when combining MLPA and NGS, underscores the urgency of early identification of DMD. Timely molecular diagnosis is essential for early treatment initiation, a strategy considered essential in hereditary neuromuscular conditions to maximize functional outcomes [45]. Although there is no absolute consensus on the very early administration of glucocorticoids, the current clinical trend supports this approach to prolong ambulation, and rapid detection facilitates this therapeutic decision. The importance of early identification also lies in the need to incorporate crucial multidisciplinary interventions (psychological, social, and family) from the moment of diagnosis [45]. Furthermore, the development of gene therapies such as delandistrogene moxeparovec, which seeks to partially restore dystrophin, necessarily requires early administration to impact the course of the disease [46]. Therefore, increasing the diagnostic accuracy of molecular methodologies directly translates into the ability to access disease-modifying treatments at the time of greatest clinical benefit.

Another issue to consider is that the recommendation of the sequential pathway (MLPA followed by NGS) directly impacts the clinical workflow by offering maximum diagnostic certainty. However, its implementation requires a detailed assessment of costs, benefits, and the burden of genetic counseling. While the high detection rate of 97% ensures the identification of most cases of dystrophy, the need to sequence two tests affects the time to diagnosis and resources, factors that are critical for initiating early treatment and mitigating the impact on the patient. Therefore, future guidelines should integrate statistical accuracy with clinical outcome metrics, such as time to therapy initiation and resource management.

### Implications of the bias and certainty in the results and applicability

The certainty of evidence was very low for MLPA specificity and moderate for its sensitivity. Lack of blinding was justified by clinical guidelines recommending sequential testing due to varying disease mechanisms [1]. Certainty for detection rates of NGS and MLPA + NGS was similarly low, leading to very limited confidence in their diagnostic performance. In addition, a significant risk of bias was identified that likely influences these pooled estimates. A high risk of bias was observed in patient selection in nearly 18% of MLPA studies and, more seriously, around flow and timing in 57% of NGS studies, as the exact timing of the test was often not specified. These methodological shortcomings, particularly the lack of blinding, which were only justified by clinical guidelines recommending sequential testing due to different disease mechanisms, may have underestimated the reported accuracy. Therefore, the direct application of our results to clinical decision-making should be interpreted with extreme caution, as real-world accuracy is likely to vary considerably depending on the specific geographic context and population. This underscores the urgent need for future prospective studies using standardized protocols to consolidate the actual diagnostic reliability of these molecular techniques. Also, high heterogeneity was reported in almost all outcomes, which could not be explained in the sensitivity analysis, implying that the heterogeneity is not due to specific studies but probably originates from intrinsic differences in all studies (e.g., differences in DNA extraction methods, diagnostic confirmation criteria, among others), which should be considered when interpreting our results and should be taken into account when conducting future studies on the topic.

### Limitations potential biases and strengths in the review process

This study has several limitations that should be mentioned. First, the included studies varied in terms of patient populations, sample sizes, and methodological approaches, which can introduce variability in the results and limit the generalizability of our findings. Second, some studies exhibited a high risk of bias in patient selection or flow and timing, which can affect the reliability of their results. Third, while the combined sample size is relatively large, individual studies may have limited sample sizes, potentially affecting the precision of the estimates. Fourth, our focus on detection rate may not fully capture the clinical impact of these diagnostic tests. Fifth, the ability of MLPA and NGS to detect specific types of mutations may vary. Sixth, gray literature such as Google Scholar was not reviewed. Seventh, given that most studies were conducted in Asia, this is likely to limit the generalization of our results to more global contexts. Eighth, publication bias was reported for the diagnostic accuracy of MLPA, so this result should be viewed with caution as it may overestimate its true effect in clinical practice. Finally, the field of genetic testing is rapidly evolving, and newer technologies may emerge with improved performance and reduced costs. On the other hand, it also has strengths. First, not only was an exhaustive search conducted in various databases, but the diagnostic utility of the tests was evaluated both individually and in combination. Second, a sensitivity analysis was performed to assess the robustness of our results and evaluate possible sources of heterogeneity. Finally, an analysis of the certainty of the evidence was performed to ensure an adequate interpretation of our results.

## Conclusions

In patients with clinical suspicion of DMD, the MLPA test has moderate sensitivity and good specificity to establish the diagnosis but with very low certainty. It can present moderately false negative results. In patients with clinical suspicion of DMD but negative MLPA results, the addition of NGS, substantially improves the detection rate. These results are consistent with the recommendations of international guidelines on the molecular genetic approach in patients with DMD/BMD; however, the low certainty of the evidence in almost all results would indicate that future research is needed to address the shortcomings of the studies in order to achieve a better interpretation of the results.

## Supporting information

S1 File S1 Table. Checklist of PRISMA-DTA.S2 Table. Search strategy for both clinical questions. S3 Table. Decision table based on GRADE system (Adaptation by authors).S4 Table. List of articles excluded. S5 Table. Quality of the studies on the use of MLPA in patients with clinical suspicion of DMD/BMD. S6 Table. Quality of the studies selected on the use of NGS in patients with clinical suspicion of DMD/ BMD. S7 Table. Brief review about cost-effective comparison between MLPA and NGS testing. S1 Fig. Deek’s funnel plot for MLPA diagnostic precision. S2 Fig. Funnel plot for the diagnostic detection rate of NGS. S3 Fig. Sensitivity analysis for MLPA diagnostic accuracy. A) Sensitivity; B) Specificity. S4 Fig. Sensitivity analysis for detection rate. A) NGS; B) MPLA + NGS.
(DOCX)
