# Supplementary material for: Accuracy of the molecular diagnosis of duchenne and becker muscular dystrophy: A systematic review with meta-analysis
Source: PLoS One. 2026 Jul 21;21(7):e0345550. doi: 10.1371/journal.pone.0345550 (PMC13387558; doi:10.1371/journal.pone.0345550)
Supplement: S1 File — Checklist of PRISMA-DTA.S2 Table. Search strategy for both clinical questions. S3 Table. Decision table based on GRADE system (Adaptation by authors).S4 Table. List of articles excluded. S5 Table. Quality of the studies on the use of MLPA in patients with clinical suspicion of DMD/BMD. S6 Table. Quality of the studies selected on the use of NGS in patients with clinical suspicion of DMD/ BMD. S7 Table. Brief review about cost-effective comparison between MLPA and NGS testing. S1 Fig. Deek’s funnel plot for MLPA diagnostic precision. S2 Fig. Funnel plot for the diagnostic detection rate of NGS. S3 Fig. Sensitivity analysis for MLPA diagnostic accuracy. A) Sensitivity; B) Specificity. S4 Fig. Sensitivity analysis for detection rate. A) NGS; B) MPLA + NGS. (DOCX) [file pone.0345550.s001.docx]

**Supplementary Material**

**Supplementary Table 1. Checklist of PRISMA-DTA**

**Supplementary Table 2. Search strategy for both clinical questions**

**Supplementary Table 3. Decision table based on GRADE system (Adaptation by authors).**

**Supplementary Table 4. List of articles excluded**

**Supplementary Table 5. Quality of the studies on the use of MLPA in patients with clinical suspicion of DMD / BMD**

**Supplementary Table 6. Quality of the studies selected on the use of NGS in patients with clinical suspicion of DMD / BMD**

**Supplementary Table 7. Brief review about cost-effective comparison between MLPA and NGS testing.**

**Supplementary Figure 1. Deek’s funnel plot for MLPA diagnostic precision**

**Supplementary Figure 2. Funnel plot for the diagnostic detection rate of NGS**

**Supplementary Figure 3. Sensitivity analysis for MLPA diagnostic accuracy. A) Sensitivity; B) Specificity**

**Supplementary Figure 4. Sensitivity analysis for detection rate. A) NGS; B) MPLA + NGS**
