# Supplementary material for: Accuracy of the molecular diagnosis of duchenne and becker muscular dystrophy: A systematic review with meta-analysis
Source: PLoS One. 2026 Jul 21;21(7):e0345550. doi: 10.1371/journal.pone.0345550 (PMC13387558; doi:10.1371/journal.pone.0345550)
Supplement: Attachment — Submitted filename: Response to Reviewers.docx [file pone.0345550.s003.docx]

**Response to reviewers**

Dear Editors,

We sincerely appreciate your review of our manuscript. In response to the reviewers' comments, we have made several revisions, which have been clearly highlighted in the manuscript. Below, we provide a detailed description of the comments along with our corresponding responses.

Carlos Alva-Diaz

**Reviewer 1**

- **Commentary 1:** “meta-analysis using a random31 effects model to estimate pooled effects was planned for each outcome.”: As it's currently worded, it sounds like it was planned but not carried out. I recommend rephrasing the sentence to state that the meta-analysis was performed.

**Reply:** Thank you for your commentary. We have made corrections.

- **Commentary 2:** “We selected 10 studies (3786 patients) evaluating”: 'selected' implies that the authors selected arbitrarily. It is suggested to change the phrase to “10 studies were included…”.

**Reply:** Thank you for your commentary. We have made corrections.

- **Commentary 3:** I2: I in italics, and 2 in superscript.

**Reply:** Thank you for your commentary. We have made corrections.

- **Commentary 4:** The introduction is well-written and coherent. As a suggestion, I would include the location of the DMD gene.

**Reply:** Thank you for your commentary. We have added the following sentence to the introduction: “gene located on the short arm of the X chromosome, at locus Xp21.2-p21.1, which encodes the dystrophin protein found in muscle cells.”

- **Commentary 5:** “following PRISMA guidelines12”: I suppose 12 should be in brackets.

**Reply:** Thank you for your commentary. We have made corrections.

- **Commentary 6:** “cross-sectional studies 108 without diagnostic outcomes,”: This criterion is redundant, since the diagnosis of DMD/BMD has already been included as an inclusion criteria.

**Reply:** Thank you for your commentary. We have made corrections.

- **Commentary 7:** “to identify studies that potentially met 125 the inclusion criteria of this review using the web application Rayyan (https://www.rayyan.ai).”. Since many readers are not familiar with AI, it is suggested to explain the function of this tool a little better, whether it is only for displaying articles or if it is used for searching articles (which could be problematic).

**Reply:** Thank you for your commentary. We have added the following sentence: “, which is a free application that allows the management of data from multiple studies from various databases, to facilitate the selection of studies.”

- **Commentary 8:** “reached a consensus by a third 128 reviewer (VVR or CAD).”. I suggest including only one third author, as including two different authors could be problematic to resolve conflicts, since it should be one or the other, but not one of the two (that creates uncertainty about how the studies were actually selected).

**Reply:** Thank you for your commentary. We have made corrections.

- **Commentary 9:** “Data sources and searches”: Were the references of the included studies reviewed? Was grey literature searched? Was an open search conducted on Google Scholar? According to AMSTAR-2, this would be advisable.

**Reply:** Thank you for your commentary. No search was conducted in gray literature or databases such as Google Scholar because priority was given to searching databases that allow for a systematic search of studies using keywords and Boolean operators.

- **Commentary 10:** “MLPA and/or NGS until 1st October 2021,”: The search is four years old; it absolutely must be updated, even if it means redoing the analyses.

**Reply:** Thank you for your commentary. Although the initial search was conducted in 2021, as described in the adjacent sentence, there was an update in July 2025.

- **Commentary 11:** The statistical analysis (Lines 162-173) requires its own section. Furthermore, what type of random effects were used? DL? Bayes? The model used must also be cited. A classification of heterogeneity is also required (citing the Cochrane).

**Reply:** Thank you for your commentary. We have made corrections in method section.

- **Commentary 12:** Also, perform an assessment of publication bias.

**Reply:** Thank you for your commentary. We have conducted a publication bias analysis.


**Reply:** Thank you for your commentary. We have made corrections in method section.

- **Commentary 14:** There shouldn't be two flowcharts, only one. Your study is a systematic review (even though it includes several meta-analyses). You must create a single flowchart (Tables 1 and 2, although they could be merged into a single table, aren't strictly necessary. But the flowchart is).

**Reply:** Thank you for your commentary. As described in more detail in the initial section on methods, this review addresses two research questions, which is why we considered it more appropriate to create two flowcharts and two tables, given that there are two different tests.

- **Commentary 15:** “Risk of bias assessment”: In addition to the percentages (18.2%, 57.1%,…), indicate the number of studies (X out of Y studies)

**Reply:** Thank you for your commentary. We have made corrections.

- **Commentary 16:** Figure 3: Try to improve the resolution a bit (I downloaded the image, obviously).

**Reply:** Thank you for your commentary. We have made corrections.

- **Commentary 17:** Figure 4: It looks strange (very dark image; only the vertical dashed line appears in normal red. I don't know if it's my computer's fault or if it wasn't exported correctly).

**Reply:** Thank you for your commentary. We have made corrections.

- **Commentary 18:** “We found that NGS had a detection rate of 73.17% for patients negative for MLPA, 316 and when combined with MLPA in a step-wise approach, it yielded a diagnosis in over 94.73% 317 of patients.”: I don't quite understand this sentence. Please rephrase it.

**Reply:** Thank you for your commentary. We have made corrections.

- **Commentary 19:** The authors should have previously assessed for publication bias. If publication bias exists, it should be discussed in the discussion.

**Reply:** Thank you for your commentary. We have conducted a publication bias analysis and discussed that result.

- **Commentary 20:** “(such as persistent 349 hyperkalemia and myopathy on the electromyogram) but without defined clinical 350 manifestations [46] to provide early diagnosis and treatment.”: Correction: the quote should be at the end of the sentence, not in the middle.

**Reply:** Thank you for your commentary. We have made corrections.

- **Commentary 21:** Regarding the above, it's necessary to expand a bit on something you've implied. You suggest that early treatment is necessary, and that's probably true. While there isn't absolute consensus on the very early administration of glucocorticoids, the trend is in that direction. Furthermore, other types of interventions (psychological, social, family-related, etc.) must also be considered. Finally, the development of delandystrogene moxeparovec, which could partially alter the course of the disease, might require early administration. Three reviews that I believe would enrich these statements are: https://pubmed.ncbi.nlm.nih.gov/38306060/ and https://pubmed.ncbi.nlm.nih.gov/38306060/ for the possibility of the need for early treatments) and https://pubmed.ncbi.nlm.nih.gov/40569438/ (for delandistrogene).

**Reply:** Thank you for your commentary. We have added the following sentence: “The high molecular diagnostic accuracy found in this review, particularly when combining MLPA and NGS, underscores the urgency of early identification of DMD. Timely molecular diagnosis is essential for early treatment initiation, a strategy considered essential in hereditary neuromuscular conditions to maximize functional outcomes [48]. Although there is no absolute consensus on the very early administration of glucocorticoids, the current clinical trend supports this approach to prolong ambulation, and rapid detection facilitates this therapeutic decision. The importance of early identification also lies in the need to incorporate crucial multidisciplinary interventions (psychological, social, and family) from the moment of diagnosis [48]. Furthermore, the development of gene therapies such as delandistrogene moxeparovec, which seeks to partially restore dystrophin, necessarily requires early administration to impact the course of the disease [49]. Therefore, increasing the diagnostic accuracy of molecular methodologies directly translates into the ability to access disease-modifying treatments at the time of greatest clinical benefit".

- **Commentary 22:** This isn't a suggestion; what I'm saying is a positive point: the PRISMA checklist was provided, as well as the list of excluded studies. This is something many authors "forget."

**Reply:** Thank you for your commentary.

**Reviewer 2**

- **Commentary 1:** First, in the abstract, the authors had better describe relevant characteristics of the studies (including the reference standard). These items are included in PRISMA-DTA for Abstracts Checklist.

**Reply:** Thank you for your commentary. We have made corrections.

- **Commentary 2:** Second, they had better revise the manuscript or add new limitations in the discussion if any important items in the PRISMA-DTA Checklist were not met.

**Reply:** Thank you for your commentary. We have made corrections.

**Reviewer 3**

- **Commentary 1:** I have no major concerns about the manuscript, but I disagree with the conclusion (lines 387-389) "Our confidence in the specificity of the MLPA and MLPA-NGS algorithm test is limited. Quality research assessing the usefulness of both tests should be performed to ensure the utility of these diagnostic interventions", as the results shown confirm the recommendations of international guidelines on the genetic molecular approach in patients with clinically suspected dystrophinopathy.

**Reply:** Thank you for your commentary. We have made corrections in conclusions.

- **Commentary 2:** Minor Concern

Line 349 : the term "hyperKalemia" should be replaced with "hyperCKemia"

**Reply:** Thank you for your commentary. We have made corrections.

**Reviewer 4**

- **Commentary 1:** The ethnic origin of the subjects in the selected studies was predominantly Asian, with limited representation from Latin America, and no studies from Europe, Africa, or North America. This sampling bias could limit the generalizability of the findings and should be highlighted as a major limitation. I find it strange that no studies from Europe or North America could be included in this meta-analysis.

**Reply:** Thank you for your commentary. We have added the limitation.

- **Commentary 2:** The overarching issue is the very low certainty of evidence for most primary outcomes. The sensitivity for MLPA and the detection rates for NGS and MLPA+NGS are all rated as "very low certainty," primarily due to substantial statistical heterogeneity (I2 > 80% for many pooled estimates), inconsistencies across the included studies due to their diverse designs and populations, and imprecision based on wide confidence intervals in some pooled estimates. This undermines confidence in the pooled diagnostic accuracy estimates, which should be explicitly stated as a limitation with more discussion on how it affects the clinical utility of the findings.

**Reply:** Thank you for your commentary. We added the following sentences “In addition, a significant risk of bias was identified that likely influences these pooled estimates. A high risk of bias was observed in patient selection in nearly 18% of MLPA studies and, more seriously, in the area of flow and timing in 57% of NGS studies, as the exact timing of the test was often not specified. These methodological shortcomings, particularly the lack of blinding, which was only justified by clinical guidelines recommending sequential testing due to different disease mechanisms, may have underestimated the reported accuracy. Therefore, the direct application of our results to clinical decision-making should be interpreted with extreme caution, as real-world accuracy is likely to vary considerably depending on the specific geographic context and population. This underscores the urgent need for future prospective studies using standardized protocols to consolidate the actual diagnostic reliability of these molecular techniques.”

- **Commentary 3:** Although QUADAS-2 was used, a high risk of bias remains in patient selection for MLPA studies (nearly 18% of the included studies) and in the flow and timing for NGS studies (57% of the studies did not specify the timing). These issues could strongly influence summary estimates and should be examined more thoroughly in the discussion.

**Reply:** Thank you for your commentary. We added the following sentences “In addition, a significant risk of bias was identified that likely influences these pooled estimates. A high risk of bias was observed in patient selection in nearly 18% of MLPA studies and, more seriously, in the area of flow and timing in 57% of NGS studies, as the exact timing of the test was often not specified. These methodological shortcomings, particularly the lack of blinding, which was only justified by clinical guidelines recommending sequential testing due to different disease mechanisms, may have underestimated the reported accuracy. Therefore, the direct application of our results to clinical decision-making should be interpreted with extreme caution, as real-world accuracy is likely to vary considerably depending on the specific geographic context and population. This underscores the urgent need for future prospective studies using standardized protocols to consolidate the actual diagnostic reliability of these molecular techniques.”

- **Commentary 4:** Focusing on detection rates and standard test performance parameters does not fully reflect the clinical impact, such as the effect on patient management or outcomes. Although the sequential diagnostic pathway (MLPA followed by NGS) is recommended, the real-world workflow implications, costs, and counseling burdens are not sufficiently addressed.

**Reply:** Thank you for your commentary. We added the following sentence “Another issue to consider is that the recommendation of the sequential pathway (MLPA followed by NGS) directly impacts the clinical workflow by offering maximum diagnostic certainty. However, its implementation requires a detailed assessment of costs, benefits, and the burden of genetic counseling. While the high detection rate of 97% ensures the identification of most cases of dystrophy, the need to sequence two tests affects the time to diagnosis and resources, factors that are critical for initiating early treatment and mitigating the impact on the patient. Therefore, future guidelines should integrate statistical accuracy with clinical outcome metrics, such as time to therapy initiation and resource management”.

- **Commentary 5:** In my opinion, the authors' conclusions (lines 384–389) are excessive and in stark contrast to the beliefs of the scientific community. I disagree with them.

**Reply:** Thank you for your commentary. We have made corrections in conclusions.

- **Commentary 6:** There are grammatical errors, awkward phrasing, and inconsistent terminology (e.g., "rare variant" vs. "mutation") in the text. A careful language revision by a fluent English speaker is strongly recommended. The manuscript could benefit from improved organization (e.g., the methods and results sections are occasionally mixed).

**Reply:** Thank you for your commentary. We have made corrections.

- **Commentary 7:** The details about cross-checking, dispute resolution, and the role of third reviewers should be presented more clearly.

**Reply:** Thank you for your commentary. We have made corrections.

- **Commentary 8:** The text briefly mentions high costs and cost-effectiveness, but lacks a structured analysis. It would be useful to include a table summarizing the cost, availability, and time-to-result for MLPA vs. NGS.

**Reply:** Thank you for your commentary. We have made corrections.
