# Supplementary material for: Accuracy of the molecular diagnosis of duchenne and becker muscular dystrophy: A systematic review with meta-analysis
Source: PLoS One. 2026 Jul 21;21(7):e0345550. doi: 10.1371/journal.pone.0345550 (PMC13387558; doi:10.1371/journal.pone.0345550)
Supplement: Peer review history

# Peer review: Decision Letter 0

**DOI:** 10.1371/journal.pone.0345550.r001

**Authors:** Tai-Heng Chen

20 Nov 2025

PONE-D-25-45832

Accuracy of the Molecular Diagnosis of Duchenne and Becker Muscular Dystrophy: A Systematic Review with Meta-analysis

PLOS ONE

Dear Dr. Alva-Diaz,

Thank you for submitting your manuscript to PLOS ONE. After careful consideration, we feel that it has merit but does not fully meet PLOS ONE’s publication criteria as it currently stands. Therefore, we invite you to submit a revised version of the manuscript that addresses the points raised during the review process.

Please submit your revised manuscript by Jan 04 2026 11:59PM. If you will need more time than this to complete your revisions, please reply to this message or contact the journal office at plosone@plos.org. When you're ready to submit your revision, log on to https://www.editorialmanager.com/pone/ and select the 'Submissions Needing Revision' folder to locate your manuscript file.

Please include the following items when submitting your revised manuscript:

- A rebuttal letter that responds to each point raised by the academic editor and reviewer(s). You should upload this letter as a separate file labeled 'Response to Reviewers'.
- A marked-up copy of your manuscript that highlights changes made to the original version. You should upload this as a separate file labeled 'Revised Manuscript with Track Changes'.
- An unmarked version of your revised paper without tracked changes. You should upload this as a separate file labeled 'Manuscript'.

If you would like to make changes to your financial disclosure, please include your updated statement in your cover letter. Guidelines for resubmitting your figure files are available below the reviewer comments at the end of this letter.

If applicable, we recommend that you deposit your laboratory protocols in protocols.io to enhance the reproducibility of your results. Protocols.io assigns your protocol its own identifier (DOI) so that it can be cited independently in the future. For instructions see: https://journals.plos.org/plosone/s/submission-guidelines#loc-laboratory-protocols. Additionally, PLOS ONE offers an option for publishing peer-reviewed Lab Protocol articles, which describe protocols hosted on protocols.io. Read more information on sharing protocols at https://plos.org/protocols?utm_medium=editorial-email&utm_source=authorletters&utm_campaign=protocols.

We look forward to receiving your revised manuscript.

Kind regards,

Tai-Heng Chen, M.D., Ph.D.

Academic Editor

PLOS ONE

Journal Requirements:

When submitting your revision, we need you to address these additional requirements.

1. Please ensure that your manuscript meets PLOS ONE's style requirements, including those for file naming. The PLOS ONE style templates can be found at

https://journals.plos.org/plosone/s/file?id=wjVg/PLOSOne_formatting_sample_main_body.pdf and

https://journals.plos.org/plosone/s/file?id=ba62/PLOSOne_formatting_sample_title_authors_affiliations.pdf

2. PLOS requires an ORCID iD for the corresponding author in Editorial Manager on papers submitted after December 6th, 2016. Please ensure that you have an ORCID iD and that it is validated in Editorial Manager. To do this, go to ‘Update my Information’ (in the upper left-hand corner of the main menu), and click on the Fetch/Validate link next to the ORCID field. This will take you to the ORCID site and allow you to create a new iD or authenticate a pre-existing iD in Editorial Manager.

3. Please amend your list of authors on the manuscript to ensure that each author is linked to an affiliation. Authors’ affiliations should reflect the institution where the work was done (if authors moved subsequently, you can also list the new affiliation stating “current affiliation:….” as necessary).

4. Please upload a new copy of Figure 4 as the detail is not clear. Please follow the link for more information:  https://journals.plos.org/plosone/s/figures

5. If the reviewer comments include a recommendation to cite specific previously published works, please review and evaluate these publications to determine whether they are relevant and should be cited. There is no requirement to cite these works unless the editor has indicated otherwise.

Reviewers' comments:

Reviewer's Responses to Questions

**Comments to the Author**

1. Is the manuscript technically sound, and do the data support the conclusions?

The manuscript must describe a technically sound piece of scientific research with data that supports the conclusions. Experiments must have been conducted rigorously, with appropriate controls, replication, and sample sizes. The conclusions must be drawn appropriately based on the data presented.

Reviewer #1: Yes

Reviewer #2: Yes

Reviewer #3: Yes

Reviewer #4: Partly

**********

2. Has the statistical analysis been performed appropriately and rigorously?

Reviewer #1: Yes

Reviewer #2: Yes

Reviewer #3: I Don't Know

Reviewer #4: Yes

**********

3. Have the authors made all data underlying the findings in their manuscript fully available?

The PLOS Data policy requires authors to make all data underlying the findings described in their manuscript fully available without restriction, with rare exception (please refer to the Data Availability Statement in the manuscript PDF file). The data should be provided as part of the manuscript or its supporting information, or deposited to a public repository. For example, in addition to summary statistics, the data points behind means, medians and variance measures should be available. If there are restrictions on publicly sharing data—e.g. participant privacy or use of data from a third party—those must be specified.

Reviewer #1: Yes

Reviewer #2: Yes

Reviewer #3: Yes

Reviewer #4: Yes

**********

4. Is the manuscript presented in an intelligible fashion and written in standard English?

PLOS ONE does not copyedit accepted manuscripts, so the language in submitted articles must be clear, correct, and unambiguous. Any typographical or grammatical errors should be corrected at revision, so please note any specific errors here.

Reviewer #1: Yes

Reviewer #2: Yes

Reviewer #3: Yes

Reviewer #4: Yes

**********

5. Review Comments to the Author

Please use the space provided to explain your answers to the questions above. You may also include additional comments for the author, including concerns about dual publication, research ethics, or publication ethics. (Please upload your review as an attachment if it exceeds 20,000 characters)

Reviewer #1: Comments to authors

This study assess the diagnostic accuracy of the molecular techniques MLPA and NGS for detecting Duchenne and Becker muscular dystrophies. It concludes that the sequential use of MLPA followed by NGS significantly improves the detection rate. Despite its potential, it requires some improvements, as well as an updated search strategy.

Abstract

• “meta-analysis using a random31 effects model to estimate pooled effects was planned for each outcome.”: As it's currently worded, it sounds like it was planned but not carried out. I recommend rephrasing the sentence to state that the meta-analysis was performed.

• “We selected 10 studies (3786 patients) evaluating”: 'selected' implies that the authors selected arbitrarily. It is suggested to change the phrase to “10 studies were included…”.

• I2: I in italics, and 2 in superscript.

Introduction

• The introduction is well-written and coherent. As a suggestion, I would include the location of the DMD gene.

Methods

• “following PRISMA guidelines12”: I suppose 12 should be in brackets.

• “cross-sectional studies 108 without diagnostic outcomes,”: This criterion is redundant, since the diagnosis of DMD/BMD has already been included as an inclusion criteria.

• “to identify studies that potentially met 125 the inclusion criteria of this review using the web application Rayyan (https://www.rayyan.ai).”. Since many readers are not familiar with AI, it is suggested to explain the function of this tool a little better, whether it is only for displaying articles or if it is used for searching articles (which could be problematic).

• “reached a consensus by a third 128 reviewer (VVR or CAD).”. I suggest including only one third author, as including two different authors could be problematic to resolve conflicts, since it should be one or the other, but not one of the two (that creates uncertainty about how the studies were actually selected).

• “Data sources and searches”: Were the references of the included studies reviewed? Was grey literature searched? Was an open search conducted on Google Scholar? According to AMSTAR-2, this would be advisable.

• “MLPA and/or NGS until 1st October 2021,”: The search is four years old; it absolutely must be updated, even if it means redoing the analyses.

• The statistical analysis (Lines 162-173) requires its own section. Furthermore, what type of random effects were used? DL? Bayes? The model used must also be cited. A classification of heterogeneity is also required (citing the Cochrane).

• Also, perform an assessment of publication bias.

• The GRADE assessment must be completed before the statistical analyses.

Results

• There shouldn't be two flowcharts, only one. Your study is a systematic review (even though it includes several meta-analyses). You must create a single flowchart (Tables 1 and 2, although they could be merged into a single table, aren't strictly necessary. But the flowchart is).

• “Risk of bias assessment”: In addition to the percentages (18.2%, 57.1%,…), indicate the number of studies (X out of Y studies).

• Figure 3: Try to improve the resolution a bit (I downloaded the image, obviously).

• Figure 4: It looks strange (very dark image; only the vertical dashed line appears in normal red. I don't know if it's my computer's fault or if it wasn't exported correctly).

Discussion

• “We found that NGS had a detection rate of 73.17% for patients negative for MLPA, 316 and when combined with MLPA in a step-wise approach, it yielded a diagnosis in over 94.73% 317 of patients.”: I don't quite understand this sentence. Please rephrase it.

• The authors should have previously assessed for publication bias. If publication bias exists, it should be discussed in the discussion.

• “(such as persistent 349 hyperkalemia and myopathy on the electromyogram) but without defined clinical 350 manifestations [46] to provide early diagnosis and treatment.”: Correction: the quote should be at the end of the sentence, not in the middle.

• Regarding the above, it's necessary to expand a bit on something you've implied. You suggest that early treatment is necessary, and that's probably true. While there isn't absolute consensus on the very early administration of glucocorticoids, the trend is in that direction. Furthermore, other types of interventions (psychological, social, family-related, etc.) must also be considered. Finally, the development of delandystrogene moxeparovec, which could partially alter the course of the disease, might require early administration. Three reviews that I believe would enrich these statements are: https://pubmed.ncbi.nlm.nih.gov/38306060/ and https://pubmed.ncbi.nlm.nih.gov/38306060/ for the possibility of the need for early treatments) and https://pubmed.ncbi.nlm.nih.gov/40569438/ (for delandistrogene).

Others

• This isn't a suggestion; what I'm saying is a positive point: the PRISMA checklist was provided, as well as the list of excluded studies. This is something many authors "forget."

Reviewer #2: This study aimed to evaluate the diagnostic accuracy of multiplex ligation-dependent probe amplification (MLPA), Next Generation Sequencing (NGS), and the algorithm MLPA-NGS for confirmatory diagnosis of Duchenne Muscular Dystrophy/Becker Muscular Dystrophy. The strength of this study was to conduct analysis using a rigorous method of systematic reviews. However, there were some concerns in this study.

First, in the abstract, the authors had better describe relevant characteristics of the studies (including the reference standard). These items are included in PRISMA-DTA for Abstracts Checklist.

Second, they had better revise the manuscript, or add new limitations in the discussion if any important items in the PRISMA-DTA Checklist were not met.

Reviewer #3: The authors performed a systematic review with meta-analysis of the diagnostic accuracy of dystrophinopathy, analyzing the results obtained through the two most frequently analyzed methods, such as MLPA and NGS.

They found that, when dystrophinopathy was clinically suspected, the first test performed was MPLA, which has a detection rate of approximately 65-80%. However, it has a high incidence of false negatives. For MLPA, the sensitivity and specificity were 0.80 (95% CI: 0.76-0.84; I2: 86.48) and 0.93 (95% CI: 0.87-0.96; I2: 47.27), respectively. In MLPA-negative cases, combining MLPA with NGS technique, the detection rate increased to approximately 95% of patients, although misdiagnosed cases persisted.

I have no major concerns about the manuscript, but I disagree with the conclusion (lines 387-389) "Our confidence in the specificity of the MLPA and MLPA-NGS algorithm test is limited. Quality research assessing the usefulness of both tests should be performed to ensure the utility of these diagnostic interventions", as the results shown confirm the recommendations of international guidelines on the genetic molecular approach in patients with clinically suspected dystrophinopathy.

Minor Concern

- Line 349 : the term "hyperKalemia" should be replaced with "hyperCKemia"

Reviewer #4: The authors propose a systematic review and meta-analysis to evaluate the accuracy of molecular diagnostic approaches, specifically MLPA and NGS, as well as their sequential use, in DMD/BMD diagnosis. This methodological approach has been used worldwide for a long time and represents the gold standard for DMD/BMD molecular diagnosis. Therefore, I had difficulty identifying how the authors' results and conclusions could enrich our knowledge in the field. In my opinion, the manuscript raises serious concerns and criticisms that must be addressed.

Major criticisms:

1. The ethnic origin of the subjects in the selected studies was predominantly Asian, with limited representation from Latin America, and no studies from Europe, Africa, or North America. This sampling bias could limit the generalizability of the findings and should be highlighted as a major limitation. I find it strange that no studies from Europe or North America could be included in this meta-analysis.

2. The overarching issue is the very low certainty of evidence for most primary outcomes. The sensitivity for MLPA and the detection rates for NGS and MLPA+NGS are all rated as "very low certainty," primarily due to substantial statistical heterogeneity (I2 > 80% for many pooled estimates), inconsistencies across the included studies due to their diverse designs and populations, and imprecision based on wide confidence intervals in some pooled estimates. This undermines confidence in the pooled diagnostic accuracy estimates, which should be explicitly stated as a limitation with more discussion on how it affects the clinical utility of the findings.

3. Although QUADAS-2 was used, a high risk of bias remains in patient selection for MLPA studies (nearly 18% of the included studies) and in the flow and timing for NGS studies (57% of the studies did not specify the timing). These issues could strongly influence summary estimates and should be examined more thoroughly in the discussion.

4. Focusing on detection rates and standard test performance parameters does not fully reflect the clinical impact, such as the effect on patient management or outcomes. Although the sequential diagnostic pathway (MLPA followed by NGS) is recommended, the real-world workflow implications, costs, and counseling burdens are not sufficiently addressed.

5. In my opinion, the authors' conclusions (lines 384–389) are excessive and in stark contrast to the beliefs of the scientific community. I disagree with them.

Minor criticisms:

1. There are grammatical errors, awkward phrasing, and inconsistent terminology (e.g., "rare variant" vs. "mutation") in the text. A careful language revision by a fluent English speaker is strongly recommended. The manuscript could benefit from improved organization (e.g., the methods and results sections are occasionally mixed).

2. The details about cross-checking, dispute resolution, and the role of third reviewers should be presented more clearly.

3. The text briefly mentions high costs and cost-effectiveness, but lacks a structured analysis. It would be useful to include a table summarizing the cost, availability, and time-to-result for MLPA vs. NGS.

**********

6. PLOS authors have the option to publish the peer review history of their article (what does this mean?). If published, this will include your full peer review and any attached files.

If you choose “no”, your identity will remain anonymous but your review may still be made public.

**Do you want your identity to be public for this peer review?**  For information about this choice, including consent withdrawal, please see our Privacy Policy.

Reviewer #1: No

Reviewer #2: **Yes:**  Masahiro Banno, MD, PhD

Reviewer #3: No

Reviewer #4: No

**********

[NOTE: If reviewer comments were submitted as an attachment file, they will be attached to this email and accessible via the submission site. Please log into your account, locate the manuscript record, and check for the action link "View Attachments". If this link does not appear, there are no attachment files.]

To ensure your figures meet our technical requirements, please review our figure guidelines: https://journals.plos.org/plosone/s/figures

You may also use PLOS’s free figure tool, NAAS, to help you prepare publication quality figures: https://journals.plos.org/plosone/s/figures#loc-tools-for-figure-preparation.

NAAS will assess whether your figures meet our technical requirements by comparing each figure against our figure specifications.

# Author comment: Author response to Decision Letter 1

**DOI:** 10.1371/journal.pone.0345550.r002

8 Jan 2026

Response to reviewers

Dear Editors,

We sincerely appreciate your review of our manuscript. In response to the reviewers' comments, we have made several revisions, which have been clearly highlighted in the manuscript. Below, we provide a detailed description of the comments along with our corresponding responses.

Carlos Alva-Diaz

Reviewer 1

• Commentary 1: “meta-analysis using a random31 effects model to estimate pooled effects was planned for each outcome.”: As it's currently worded, it sounds like it was planned but not carried out. I recommend rephrasing the sentence to state that the meta-analysis was performed.

Reply: Thank you for your commentary. We have made corrections.

• Commentary 2: “We selected 10 studies (3786 patients) evaluating”: 'selected' implies that the authors selected arbitrarily. It is suggested to change the phrase to “10 studies were included…”.

Reply: Thank you for your commentary. We have made corrections.

• Commentary 3: I2: I in italics, and 2 in superscript.

Reply: Thank you for your commentary. We have made corrections.

• Commentary 4: The introduction is well-written and coherent. As a suggestion, I would include the location of the DMD gene.

Reply: Thank you for your commentary. We have added the following sentence to the introduction: “gene located on the short arm of the X chromosome, at locus Xp21.2-p21.1, which encodes the dystrophin protein found in muscle cells.”

• Commentary 5: “following PRISMA guidelines12”: I suppose 12 should be in brackets.

Reply: Thank you for your commentary. We have made corrections.

• Commentary 6: “cross-sectional studies 108 without diagnostic outcomes,”: This criterion is redundant, since the diagnosis of DMD/BMD has already been included as an inclusion criteria.

Reply: Thank you for your commentary. We have made corrections.

• Commentary 7: “to identify studies that potentially met 125 the inclusion criteria of this review using the web application Rayyan (https://www.rayyan.ai).”. Since many readers are not familiar with AI, it is suggested to explain the function of this tool a little better, whether it is only for displaying articles or if it is used for searching articles (which could be problematic).

Reply: Thank you for your commentary. We have added the following sentence: “, which is a free application that allows the management of data from multiple studies from various databases, to facilitate the selection of studies.”

• Commentary 8: “reached a consensus by a third 128 reviewer (VVR or CAD).”. I suggest including only one third author, as including two different authors could be problematic to resolve conflicts, since it should be one or the other, but not one of the two (that creates uncertainty about how the studies were actually selected).

Reply: Thank you for your commentary. We have made corrections.

• Commentary 9: “Data sources and searches”: Were the references of the included studies reviewed? Was grey literature searched? Was an open search conducted on Google Scholar? According to AMSTAR-2, this would be advisable.

Reply: Thank you for your commentary. No search was conducted in gray literature or databases such as Google Scholar because priority was given to searching databases that allow for a systematic search of studies using keywords and Boolean operators.

• Commentary 10: “MLPA and/or NGS until 1st October 2021,”: The search is four years old; it absolutely must be updated, even if it means redoing the analyses.

Reply: Thank you for your commentary. Although the initial search was conducted in 2021, as described in the adjacent sentence, there was an update in July 2025.

• Commentary 11: The statistical analysis (Lines 162-173) requires its own section. Furthermore, what type of random effects were used? DL? Bayes? The model used must also be cited. A classification of heterogeneity is also required (citing the Cochrane).

Reply: Thank you for your commentary. We have made corrections in method section.

• Commentary 12: Also, perform an assessment of publication bias.

Reply: Thank you for your commentary. We have conducted a publication bias analysis.

Reply: Thank you for your commentary. We have made corrections in method section.

• Commentary 14: There shouldn't be two flowcharts, only one. Your study is a systematic review (even though it includes several meta-analyses). You must create a single flowchart (Tables 1 and 2, although they could be merged into a single table, aren't strictly necessary. But the flowchart is).

Reply: Thank you for your commentary. As described in more detail in the initial section on methods, this review addresses two research questions, which is why we considered it more appropriate to create two flowcharts and two tables, given that there are two different tests.

• Commentary 15: “Risk of bias assessment”: In addition to the percentages (18.2%, 57.1%,…), indicate the number of studies (X out of Y studies)

Reply: Thank you for your commentary. We have made corrections.

• Commentary 16: Figure 3: Try to improve the resolution a bit (I downloaded the image, obviously).

Reply: Thank you for your commentary. We have made corrections.

• Commentary 17: Figure 4: It looks strange (very dark image; only the vertical dashed line appears in normal red. I don't know if it's my computer's fault or if it wasn't exported correctly).

Reply: Thank you for your commentary. We have made corrections.

• Commentary 18: “We found that NGS had a detection rate of 73.17% for patients negative for MLPA, 316 and when combined with MLPA in a step-wise approach, it yielded a diagnosis in over 94.73% 317 of patients.”: I don't quite understand this sentence. Please rephrase it.

Reply: Thank you for your commentary. We have made corrections.

• Commentary 19: The authors should have previously assessed for publication bias. If publication bias exists, it should be discussed in the discussion.

Reply: Thank you for your commentary. We have conducted a publication bias analysis and discussed that result.

• Commentary 20: “(such as persistent 349 hyperkalemia and myopathy on the electromyogram) but without defined clinical 350 manifestations [46] to provide early diagnosis and treatment.”: Correction: the quote should be at the end of the sentence, not in the middle.

Reply: Thank you for your commentary. We have made corrections.

• Commentary 21: Regarding the above, it's necessary to expand a bit on something you've implied. You suggest that early treatment is necessary, and that's probably true. While there isn't absolute consensus on the very early administration of glucocorticoids, the trend is in that direction. Furthermore, other types of interventions (psychological, social, family-related, etc.) must also be considered. Finally, the development of delandystrogene moxeparovec, which could partially alter the course of the disease, might require early administration. Three reviews that I believe would enrich these statements are: https://pubmed.ncbi.nlm.nih.gov/38306060/ and https://pubmed.ncbi.nlm.nih.gov/38306060/ for the possibility of the need for early treatments) and https://pubmed.ncbi.nlm.nih.gov/40569438/ (for delandistrogene).

Reply: Thank you for your commentary. We have added the following sentence: “The high molecular diagnostic accuracy found in this review, particularly when combining MLPA and NGS, underscores the urgency of early identification of DMD. Timely molecular diagnosis is essential for early treatment initiation, a strategy considered essential in hereditary neuromuscular conditions to maximize functional outcomes [48]. Although there is no absolute consensus on the very early administration of glucocorticoids, the current clinical trend supports this approach to prolong ambulation, and rapid detection facilitates this therapeutic decision. The importance of early identification also lies in the need to incorporate crucial multidisciplinary interventions (psychological, social, and family) from the moment of diagnosis [48]. Furthermore, the development of gene therapies such as delandistrogene moxeparovec, which seeks to partially restore dystrophin, necessarily requires early administration to impact the course of the disease [49]. Therefore, increasing the diagnostic accuracy of molecular methodologies directly translates into the ability to access disease-modifying treatments at the time of greatest clinical benefit".

• Commentary 22: This isn't a suggestion; what I'm saying is a positive point: the PRISMA checklist was provided, as well as the list of excluded studies. This is something many authors "forget."

Reply: Thank you for your commentary.

Reviewer 2

• Commentary 1: First, in the abstract, the authors had better describe relevant characteristics of the studies (including the reference standard). These items are included in PRISMA-DTA for Abstracts Checklist.

Reply: Thank you for your commentary. We have made corrections.

• Commentary 2: Second, they had better revise the manuscript or add new limitations in the discussion if any important items in the PRISMA-DTA Checklist were not met.

Reply: Thank you for your commentary. We have made corrections.

Reviewer 3

• Commentary 1: I have no major concerns about the manuscript, but I disagree with the conclusion (lines 387-389) "Our confidence in the specificity of the MLPA and MLPA-NGS algorithm test is limited. Quality research assessing the usefulness of both tests should be performed to ensure the utility of these diagnostic interventions", as the results shown confirm the recommendations of international guidelines on the genetic molecular approach in patients with clinically suspected dystrophinopathy.

Reply: Thank you for your commentary. We have made corrections in conclusions.

• Commentary 2: Minor Concern

Line 349 : the term "hyperKalemia" should be replaced with "hyperCKemia"

Reply: Thank you for your commentary. We have made corrections.

Reviewer 4

• Commentary 1: The ethnic origin of the subjects in the selected studies was predominantly Asian, with limited representation from Latin America, and no studies from Europe, Africa, or North America. This sampling bias could limit the generalizability of the findings and should be highlighted as a major limitation. I find it strange that no studies from Europe or North America could be included in this meta-analysis.

Reply: Thank you for your commentary. We have added the limitation.

• Commentary 2: The overarching issue is the very low certainty of evidence for most primary outcomes. The sensitivity for MLPA and the detection rates for NGS and MLPA+NGS are all rated as "very low certainty," primarily due to substantial statistical heterogeneity (I2 > 80% for many pooled estimates), inconsistencies across the included studies due to their diverse designs and populations, and imprecision based on wide confidence intervals in some pooled estimates. This undermines confidence in the pooled diagnostic accuracy estimates, which should be explicitly stated as a limitation with more discussion on how it affects the clinical utility of the findings.

Reply: Thank you for your commentary. We added the following sentences “In addition, a significant risk of bias was identified that likely influences these pooled estimates. A high risk of bias was observed in patient selection in nearly 18% of MLPA studies and, more seriously, in the area of flow and timing in 57% of NGS studies, as the exact timing of the test was often not specified. These methodological shortcomings, particularly the lack of blinding, which was only justified by clinical guidelines recommending sequential testing due to different disease mechanisms, may have underestimated the reported accuracy. Therefore, the direct application of our results to clinical decision-making should be interpreted with extreme caution, as real-world accuracy is likely to vary considerably depending on the specific geographic context and population. This underscores the urgent need for future prospective studies using standardized protocols to consolidate the actual diagnostic reliability of these molecular techniques.”

• Commentary 3: Although QUADAS-2 was used, a high risk of bias remains in patient selection for MLPA studies (nearly 18% of the included studies) and in the flow and timing for NGS studies (57% of the studies did not specify the timing). These issues could strongly influence summary estimates and should be examined more thoroughly in the discussion.

Reply: Thank you for your commentary. We added the following sentences “In addition, a significant risk of bias was identified that likely influences these pooled estimates. A high risk of bias was observed in patient selection in nearly 18% of MLPA studies and, more seriously, in the area of flow and timing in 57% of NGS studies, as the exact timing of the test was often not specified. These methodological shortcomings, particularly the lack of blinding, which was only justified by clinical guidelines recommending sequential testing due to different disease mechanisms, may have underestimated the reported accuracy. Therefore, the direct application of our results to clinical decision-making should be interpreted with extreme caution, as real-world accuracy is likely to vary considerably depending on the specific geographic context and population. This underscores the urgent need for future prospective studies using standardized protocols to consolidate the actual diagnostic reliability of these molecular techniques.”

• Commentary 4: Focusing on detection rates and standard test performance parameters does not fully reflect the clinical impact, such as the effect on patient management or outcomes. Although the sequential diagnostic pathway (MLPA followed by NGS) is recommended, the real-world workflow implications, costs, and counseling burdens are not sufficiently addressed.

Reply: Thank you for your commentary. We added the following sentence “Another issue to consider is that the recommendation of the sequential pathway (MLPA followed by NGS) directly impacts the clinical workflow by offering maximum diagnostic certainty. However, its implementation requires a detailed assessment of costs, benefits, and the burden of genetic counseling. While the high detection rate of 97% ensures the identification of most cases of dystrophy, the need to sequence two tests affects the time to diagnosis and resources, factors that are critical for initiating early treatment and mitigating the impact on the patient. Therefore, future guidelines should integrate statistical accuracy with clinical outcome metrics, such as time to therapy initiation and resource management”.

• Commentary 5: In my opinion, the authors' conclusions (lines 384–389) are excessive and in stark contrast to the beliefs of the scientific community. I disagree with them.

Reply: Thank you for your commentary. We have made corrections in conclusions.

• Commentary 6: There are grammatical errors, awkward phrasing, and inconsistent terminology (e.g., "rare variant" vs. "mutation") in the text. A careful language revision by a fluent English speaker is strongly recommended. The manuscript could benefit from improved organization (e.g., the methods and results sections are occasionally mixed).

Reply: Thank you for your commentary. We have made corrections.

• Commentary 7: The details about cross-checking, dispute resolution, and the role of third reviewers should be presented more clearly.

Reply: Thank you for your commentary. We have made corrections.

• Commentary 8: The text briefly mentions high costs and cost-effectiveness, but lacks a structured analysis. It would be useful to include a table summarizing the cost, availability, and time-to-result for MLPA vs. NGS.

Reply: Thank you for your commentary. We have made corrections.

Attachment Submitted filename: Response to Reviewers.docx

# Peer review: Decision Letter 1

**DOI:** 10.1371/journal.pone.0345550.r003

**Authors:** Tai-Heng Chen, Mainak Bardhan

9 Mar 2026

Accuracy of the Molecular Diagnosis of Duchenne and Becker Muscular Dystrophy: A Systematic Review with Meta-analysis

PONE-D-25-45832R1

Dear Dr. Carlos Alva-Diaz,

We’re pleased to inform you that your manuscript has been judged scientifically suitable for publication and will be formally accepted for publication once it meets all outstanding technical requirements.

Within one week, you’ll receive an e-mail detailing the required amendments. When these have been addressed, you’ll receive a formal acceptance letter and your manuscript will be scheduled for publication.

An invoice will be generated when your article is formally accepted. Please note, if your institution has a publishing partnership with PLOS and your article meets the relevant criteria, all or part of your publication costs will be covered. Please make sure your user information is up-to-date by logging into Editorial Manager at Editorial Manager® and clicking the ‘Update My Information' link at the top of the page. For questions related to billing, please contact billing support.

If your institution or institutions have a press office, please notify them about your upcoming paper to help maximize its impact. If they’ll be preparing press materials, please inform our press team as soon as possible -- no later than 48 hours after receiving the formal acceptance. Your manuscript will remain under strict press embargo until 2 pm Eastern Time on the date of publication. For more information, please contact onepress@plos.org.

Kind regards,

Mainak Bardhan, MD

Academic Editor

PLOS One

Additional Editor Comments (optional):

Reviewers' comments:

Reviewer's Responses to Questions

**Comments to the Author**

1. If the authors have adequately addressed your comments raised in a previous round of review and you feel that this manuscript is now acceptable for publication, you may indicate that here to bypass the “Comments to the Author” section, enter your conflict of interest statement in the “Confidential to Editor” section, and submit your "Accept" recommendation.

Reviewer #1: All comments have been addressed

Reviewer #2: All comments have been addressed

Reviewer #3: All comments have been addressed

Reviewer #4: (No Response)

**********

2. Is the manuscript technically sound, and do the data support the conclusions?

The manuscript must describe a technically sound piece of scientific research with data that supports the conclusions. Experiments must have been conducted rigorously, with appropriate controls, replication, and sample sizes. The conclusions must be drawn appropriately based on the data presented.

Reviewer #1: Yes

Reviewer #2: Yes

Reviewer #3: Yes

Reviewer #4: Partly

**********

3. Has the statistical analysis been performed appropriately and rigorously?

Reviewer #1: Yes

Reviewer #2: Yes

Reviewer #3: I Don't Know

Reviewer #4: Yes

**********

4. Have the authors made all data underlying the findings in their manuscript fully available?

The PLOS Data policy requires authors to make all data underlying the findings described in their manuscript fully available without restriction, with rare exception (please refer to the Data Availability Statement in the manuscript PDF file). The data should be provided as part of the manuscript or its supporting information, or deposited to a public repository. For example, in addition to summary statistics, the data points behind means, medians and variance measures should be available. If there are restrictions on publicly sharing data—e.g. participant privacy or use of data from a third party—those must be specified.

Reviewer #1: Yes

Reviewer #2: Yes

Reviewer #3: Yes

Reviewer #4: Yes

**********

5. Is the manuscript presented in an intelligible fashion and written in standard English?

PLOS ONE does not copyedit accepted manuscripts, so the language in submitted articles must be clear, correct, and unambiguous. Any typographical or grammatical errors should be corrected at revision, so please note any specific errors here.

Reviewer #1: Yes

Reviewer #2: Yes

Reviewer #3: Yes

Reviewer #4: Yes

**********

6. Review Comments to the Author

Please use the space provided to explain your answers to the questions above. You may also include additional comments for the author, including concerns about dual publication, research ethics, or publication ethics. (Please upload your review as an attachment if it exceeds 20,000 characters)

Reviewer #1: The authors carefully addressed all of the suggested comments. Furthermore, even when no changes were made (e.g. the creation of a single flowchart), the authors provided sound justification for their decisions.

Reviewer #2: The authors have revised the manuscript extensively. I have no additional comments for the manuscript.

Reviewer #3: All my comments to the previous version of the manuscript have been addressed. I have no further comments

Reviewer #4: The authors partially addressed my concerns. They highlighted the different biases reported by the reviewers, but they did not enrich the study cohort with papers from U.S. and European laboratories. I am still perplexed by the scientific contribution of this meta-analysis.

**********

7. PLOS authors have the option to publish the peer review history of their article (what does this mean?). If published, this will include your full peer review and any attached files.

If you choose “no”, your identity will remain anonymous but your review may still be made public.

**Do you want your identity to be public for this peer review?**  For information about this choice, including consent withdrawal, please see our Privacy Policy.

Reviewer #1: No

Reviewer #2: **Yes:**  Masahiro Banno, MD, PhD

Reviewer #3: No

Reviewer #4: No

**********

# Editor report: Acceptance letter

**DOI:** 10.1371/journal.pone.0345550.r004

**Authors:** Tai-Heng Chen, Mainak Bardhan

PONE-D-25-45832R1

PLOS One

Dear Dr. Alva-Diaz,

I'm pleased to inform you that your manuscript has been deemed suitable for publication in PLOS One. Congratulations! Your manuscript is now being handed over to our production team.

At this stage, our production department will prepare your paper for publication. This includes ensuring the following:

* All references, tables, and figures are properly cited

* All relevant supporting information is included in the manuscript submission,

* There are no issues that prevent the paper from being properly typeset

You will receive further instructions from the production team, including instructions on how to review your proof when it is ready. Please keep in mind that we are working through a large volume of accepted articles, so please give us a few days to review your paper and let you know the next and final steps.

Lastly, if your institution or institutions have a press office, please let them know about your upcoming paper now to help maximize its impact. If they'll be preparing press materials, please inform our press team within the next 48 hours. Your manuscript will remain under strict press embargo until 2 pm Eastern Time on the date of publication. For more information, please contact onepress@plos.org.

You will receive an invoice from PLOS for your publication fee after your manuscript has reached the completed accept phase. If you receive an email requesting payment before acceptance or for any other service, this may be a phishing scheme. Learn how to identify phishing emails and protect your accounts at https://explore.plos.org/phishing.

If we can help with anything else, please email us at customercare@plos.org.

Thank you for submitting your work to PLOS ONE and supporting open access.

Kind regards,

PLOS ONE Editorial Office Staff

on behalf of

Dr. Mainak Bardhan

Academic Editor

PLOS One
